# Bibliography

**Published:** 1896-02

**Authors:** 


### Bibliography.

THE DISEASES OF CHILDREN'S TEETH: THEIR PREVENTION AND TREATMENT. A Manual for Medical Practitioners and Students. By R. Denison Pedley, M.R.C.S., L.D.S. (Eng.), F.R.C.S. (Edin.), Dental Surgeon to the Evelina Hospital for Sick Children, Southwark, London. With numerous illustrations. I. P. Segg & Co., London; in America, S. S. White Dental Manufacturing Company, Philadelphia, 1895.

This volume of two hundred and sixty-one pages is prepared by the author for the purpose of aiding the isolated practitioner in medicine, who, "settling down in the country where the area of practice is wide and the opportunities of obtaining skilled dental assistance are few," finds the necessity for just such advice as is contained in this book.

The opening chapter is occupied very properly with the consideration of the structure of the teeth. The tissues are described briefly, but with sufficient fulness to meet the purpose intended by the author.

In the next chapter, on "The Eruption of the Teeth," the author explains, rather too briefly, the result of the reflex disturbances observed, which change so frequently from a "physiological into a pathological process. At this period the edge of the open, forming root will be in intimate relation with the pulp-tissue, and if there is pressure by resisting gum (*vis a fronte*), the irritation may give rise to relatively the same degree of stimulus received in the case of a large exposure of pulp in an adult tooth affected by caries."

This practically answers the query, "Why lance teeth during dentition?" but as the author hopes to impress medical men, it seems to the writer he should have made this vital point more impressive, and extended it to the lesions produced by pressure upon the undeveloped region of pulp-tissue. It would have been instructive to have shown where and by what means new pathological conditions are produced.

The subsequent chapters on Caries, Pulpitis, Periodontitis are well written, and should accomplish the desired aim of instructing those needing them.

The next chapter, covering sixty-four pages, on "Irregularities of Structure," might have been condensed without materially lessening its value. It is not to be presumed that medical men will take the time necessary to study irregularities, a very difficult portion of the dentist's work,—indeed, so complicated that the treatment is gradually falling into the hands of specialists. Exception must be taken to the advice given on page 117, where the author says, "The remedy for overcrowding is judicious and timely extraction. This should be carried out between the ages of twelve and sixteen years. . . . The movements which take place in the teeth during mastication (always on the lines of least resistance) will insure the rapid filling up of gaps thus made. To remove one alone is not, however, sufficient. On referring to Fig. 28 of a normal articulation in permanent teeth, it will be seen that the bicuspids and molars interlock when the jaws are closed. The effect of removing one of these teeth alone—an upper bicuspid or molar tooth, for instance—is that the lower teeth effectually prevent the tooth on either side of the space above from approaching one another, and a gap remains. Should, however, the tooth above and its principal opponent below (as in the case of the first permanent molars) be removed, within a year's time the teeth on that side of the mouth will all have moved, the back teeth forward and perhaps the front teeth backward, *leaving an interval between each tooth.*"

This advice is directly in the line of the old teaching so firmly fastened upon the practice of forty years ago, now, however, happily relegated in this country to the obsolete methods as part of the history of dentistry. This is not the place to argue this question with the author, but it is very safe to dogmatically affirm that such methods of regulating teeth invariably produce irregularities difficult, if not impossible, to correct.

The chapter on "Oral Hygiene" the reviewer regards as the most valuable in the book. The facts therein contained should have a wider circulation than, it is feared, will be possible in book form. The author gives some of the results of the investigation of the teeth of children in parochial schools, industrial homes, and national schools made by Mr. Fisher, of Dundee, and Dr. Cunningham, of Cambridge. These reports make interesting reading. One paragraph is quoted,—"Four hundred and fifty-eight girls from pauper schools of the metropolis *alone* entered domestic service in one year. Five-sixths of that number had never known the use of a tooth-brush. . . . Referring . . . to the table of figures, it will be seen that 2972 children had among them 10,795 carious teeth."

The balance of the volume is included under the head of "Treatment," comprising treatment of toothache, stopping or filling, extraction, injuries of the teeth, and, finally, tartar and its removal.

While this book does not, as a whole, reach the standard demanded by a dental practitioner, it still is worthy an important place in his library, but it derives its greatest value as an aid in post-graduate instruction to medical practitioners.

The arrangement and work of the publisher are exceptionally good.

